# SMYD2 Promotes Hepatocellular Carcinoma Progression by Reprogramming Glutamine Metabolism via c-Myc/GLS1 Axis

**DOI:** 10.3390/cells12010025

**Published:** 2022-12-21

**Authors:** Kangdi Xu, Jun Ding, Lingfeng Zhou, Dazhi Li, Jia Luo, Wenchao Wang, Mingge Shang, Bingyi Lin, Lin Zhou, Shusen Zheng

**Affiliations:** 1 Division of Hepatobiliary and Pancreatic Surgery, Department of Surgery, The First Affiliated Hospital, Zhejiang University School of Medicine, NO.79 Qing Chun Road, Hangzhou 310006, China; 2 NHC Key Laboratory of Combined Multi-Organ Transplantation, Hangzhou 310003, China; 3 Key Laboratory of the Diagnosis and Treatment of Organ Transplantation, Research Unit of Collaborative Diagnosis and Treatment For Hepatobiliary and Pancreatic Cancer, Chinese Academy of Medical Sciences (2019RU019), Hangzhou 310003, China; 4 Key Laboratory of Organ Transplantation, Research Center for Diagnosis and Treatment of Hepatobiliary Diseases, Hangzhou 310003, China

**Keywords:** hepatocellular carcinoma, SMYD2, c-Myc, GLS1

## Abstract

Metabolic reprogramming, such as alterations in glutamine metabolism or glycolysis, is the hallmark of hepatocellular carcinoma (HCC). However, the underlying mechanisms are still incompletely elucidated. Previous studies have identified that methyltransferase SET and MYND domain-containing protein 2(SMYD2) is responsible for the pathogenesis of numerous types of cancer. Here, we innovatively uncover how SMYD2 regulates glutamine metabolism in HCC cells and promotes HCC progression. We identified that SMYD2 expression is upregulated in HCC tissues, which correlates with unfavorable clinical outcomes. Our in vitro and in vivo results showed that the depletion of SMYD2 inhibits HCC cell growth. Mechanistically, c-Myc methylation by SMYD2 increases its protein stability through the ubiquitin–proteasome system. We showed SMYD2 depletion destabilized c-Myc protein by increasing the conjugated K48-linked polyubiquitin chain. SMYD2 increased c-Myc expression and further upregulated glutaminase1 (GLS1), a crucial enzyme that catalyzes the conversion of glutamine to glutamic acid, in HCC cells. GLS1 plays an important role in SMYD2-mediated HCC progression and glutamine metabolism regulation. The knockdown of SMYD2 inhibited glutamine metabolism in HCC cells and overcame their chemoresistance to sorafenib. Collectively, our findings demonstrated a novel mechanism of how SMYD2 promotes HCC progression by regulating glutamine metabolism through the c-Myc/GLS1signaling, implicating the therapeutic potential of targeting SMYD2 in HCC patients.

## 1. Introduction

Hepatocellular carcinoma is one of the most common malignancies in the world [1,2]. Due to the lack of symptoms in the early stage, most HCC patients are diagnosed in their terminal stage [3,4]. Although much progress has been made in recent decades on the diagnosis and treatment of HCC, its prognosis remains dismal because of the poor understanding of its pathogenesis and the prevalence of increased chemoresistance. Therefore, it is urgently needed to identify the underlying mechanisms of HCC to identify the potential therapeutic target to improve its clinical outcomes.

An accumulation of evidence has shown that metabolic reprogramming is one of the hallmarks of cancer cells [5,6]. Metabolic alternation generally occurs in cancer cells to support their rapid growth. Glutamine, the most abundant amino acid in rapidly proliferating cells and the bloodstream [7,8,9,10], acts as an important carbon source for anabolic processes and energy production [11]. Glutamine metabolism converts glutamine to glutamate, which is catalyzed by glutaminase (GLS), then further enters into tricarboxylic acid (TCA) cycle metabolites to generate ATP. The glutamine-derived metabolic intermediates may also act as a shunt in the production of NAD(P)H and glutathione (GSH) to regulate cellular redox homeostasis [12]. Many new findings have highlighted the therapeutic potential of glutamine inhibition in cancer cells because of its crucial role in cell proliferation. Thus, uncovering the role of glutamine metabolism in HCC may pave the road for developing more effective therapies for HCC. Glutaminase1(GLS1), a major isoform of GLS, that catalyzes glutamine to ammonia and glutamate, to support nitrogen balance, bioenergetics, and metabolism homeostasis, was reported overexpressed in many tumors and could regulate the stemness of HCC [13,14,15]. Moreover, the oncogenic transcription factor c-MYC is reported to be able to stimulate glutamine metabolism by upregulating GLS to support the growth of cancer cells [16,17]. However, the underlying mechanisms of how c-MYC regulated GLS1 are less well recognized as of yet, and need more studies to clarify.

The protein methyltransferase SET and MYND domain-containing protein 2(SMYD2) is a histone lysine methyltransferase and is reported to act as a candidate oncogene in many tumors. SMYD2 is one of the well-recognized lysine methyltransferases, which can catalyze the methylation of lysine 4 and 36 (H3K4 and H3K36) on histones, as well as nonhistone targets. In addition, SMYD2 can promote the phosphorylation of AKT and ERK1/2 by regulating PTPN13 in breast cancer cells [18,19]. Recent studies suggested that SMYD2 promotes cancer cell’ progression by inhibiting the function of tumor suppressor proteins such as p53, PTEN, and Rb [19,20,21,22]. However, the role of SMYD2 in HCC remains elusive and to date, less is known about its function in glutamine reprogramming, which inspired us to investigate the function of SMYD2 in HCC.

In the current study, we innovatively uncovered how SMYD2 reprograms glutamine metabolism in HCC cells and promotes HCC progression. To elucidate the significance of SMYD2 in HCC, we evaluated the correlation of SMYD2 expression with HCC patients’ prognosis. Then, we unveil the role of SMYD2 in HCC progression in vitro and in xenograft mouse models. Mechanistically, we thoroughly elucidated how SMYD2 activates glutamine metabolism via the c-Myc/GLS1 axis. Furthermore, we showed targeting SMYD2 in HCC cells may inhibit glutamine metabolism and further sensitizes their response to sorafenib. Taken together, this study provides strong evidence that SMYD2 enhances HCC progression by regulating glutamine metabolism, may serve as a promising prognostic biomarker, and act as a potential therapeutic target in HCC.

## 2. Materials and Methods

### 2.1. Human Tissue Specimens

We collected 80 HCC specimens and their matched normal liver tissues from the First Affiliated Hospital, Zhejiang University School of Medicine. The Clinical Research Ethics Committee of this hospital approved this study. Written informed consent was received from all patients.

### 2.2. Cell Culture

All cell lines were obtained from the Liver Cancer Institute of Fudan University. The cells were cultured in MEM (BI, Kibbutz Beit Haemek, Israel) with 10% fetal bovine serum in a 5% CO_2_, 37 °C incubator. Cycloheximide, 10058-F4 and MG132 were obtained from MCE (Monmouth Junction, NJ, USA).

### 2.3. RNA Extraction and RT-qPCR

RNA was extracted using TRIzol reagent (Invitrogen, Waltham, MA, USA). Reverse transcription (RT) was performed by HiScript Q RT SuperMix (Vazyme, Nanjing, China). An SYBR PCR Kit (Vazyme, China) was used to conduct RT-PCR. All primers are listed in the Supplementary Materials and Methods.

### 2.4. Knockdown and Forced Expression of Targeted Genes

Specific siRNAs targeting SMYD2 were obtained from GenePharma (Shanghai, China) and transfected with jetPRIME^®^ (Illkirch, Strasbourg, France). The siRNAs used are listed in the Supplementary Material and Methods.

shRNA targeting SMYD2 was constructed followed the sequences of si-SMYD2#1 and si-SMYD2#2. The c-Myc and GLS1 overexpression lentivirus were obtained from GenePharma (Shanghai, China). The indicated cell lines were infected with lentivirus for 48 h. The transfected cells were selected with 6 μg/mL puromycin for 5 days.

### 2.5. RNA-seq

Total RNA was extracted from shSMYD2 and shNC groups of Huh7 cells using TRIzol reagent (Invitrogen Waltham, MA, USA). OE Biotech Co, Ltd. (Shanghai, China) conducted the transcriptome high-throughput sequencing.

### 2.6. Cell Viability and Colony Formation Assays

Cell viability was examined by using the CCK-8 assay (MCE). Transfected Huh7 and HCCLM3 cells (2 × 10^3^/well) were plated into 96-well plates. Then, absorbance was measured at 450 nm. In addition, an EdU Apollo 567 kit (RiboBio, Guangzhou, China) was used to conduct the ethynyl deoxyuridine(EdU) assay.

For colony formation assays, transfected cells (1 × 10^3^/well) were plated into six-well plates in a humidified atmosphere with 5% CO_2_ at 37 °C. Cells were stained and photographed after 14 days.

### 2.7. LC-MS

The samples were desalted with STAGE from 3M Empore extraction disks. Next, they were re-dried. All samples were resuspended in an injection buffer and analyzed using liquid chromatography–tandem mass spectrometry (LC-MS).

### 2.8. Flow Cytometry Analysis

The cells were collected and fixed in 75% ethanol at −20 °C for 2 days. Then, cells were stained with DNA staining solution (Multi Sciences, Hangzhou, China). Finally, the cell cycle was detected with the BD FACSCanto^TM^II (USA).

Apoptosis was assessed using the Annexin V-APC/7-AAD Apoptosis kit (Multi Sciences, Hangzhou, China) according to the protocols. Cells were analyzed by flow cytometry.

### 2.9. Glutamate Assays and Measurement of Glutamine Consumption

Intracellular glutamate was detected by Glutamate Kit (Sigma, MO, USA), according to the manufacturer’s instructions. The total protein concentration was used to normalize the data.

An amount of 5 × 10^4^ cells were plated into a two-well plate. After 24 h incubation, the Glutamine Detection Kit (Abnova, Boston, MA, USA) was used to detect the glutamine concentrations. A blank well was used as the control.

### 2.10. Western Blotting and Co-Immunoprecipitation (Co-IP)

Cells were lysed by RIPA. The total protein concentration was examined using the BCA Kit (Thermo Scientific, Waltham, MA, USA). An amount of 40 mg of protein was loaded on ExpressPLUSTMPAGE gels (GenScript, Nanjing, China) and transferred onto PVDF membranes. These membranes were incubated with primary antibodies at 4 °C for over 12 h. Then, the membranes were incubated with HRP-conjugated secondary antibodies. The immunoblot was detected by EZ-ECL (Thermo Scientific, Waltham, MA, USA).

For the coimmunoprecipitation (co-IP) analysis, cells were treated with 10 mM MG132 (MCE, Monmouth Junction, NJ, USA) for 4–6 h. The total proteins were extracted and immunoprecipitated with primary antibodies on beads (Thermo Scientific, Waltham, MA, USA) for over 12 h. The precipitates were detected by Western blotting. The antibodies used are presented in the Supplementary Materials and Methods.

### 2.11. ChIP-qPCR Assays

This procedure was conducted by the ChIP Magnetic kit (Thermo Scientific, Waltham, MA, USA). The cell lysates were immunoprecipitated with IgG or anti-c-Myc antibody, then the purified DNA was analyzed by qPCR. Primer sequences used are shown in the Supplementary Materials and Methods.

### 2.12. Luciferase Reporter Assay

The indicated cells (2 × 10^5^/well) were seeded into 24-well plates and then transfected with 2 mg of promoter–luciferase plasmids. After 24 h post-transfection, the luciferase activity was detected by the Dual-luciferase Kit (Promega, Madison, WI, USA). The transfection efficiency was normalized to the Renilla luciferase activity.

### 2.13. Immunohistochemistry (IHC)

The immunohistochemistry was then conducted as previously described [23]. All antibodies used were shown in the Supplementary Materials and Methods.

### 2.14. Mouse Xenograft Assay

The Animal Experimental Ethics Committee of the First Affiliated Hospital of Zhejiang University School of Medicine approved the animal experiments.

The male BALB/C nude mice were obtained from Shanghai Experimental Animal Center. The mice were divided into three groups (5 mice/group) and 4 × 10^6^ HCC cells per mouse were subcutaneously injected. All mice were sacrificed and tumors were harvested for volume and weight measurement after four weeks.

The equation: tumor volume = (length × width^2^)/2 was applied to calculate the tumor volume. All antibodies used were listed in the Supplementary Materials and Methods.

### 2.15. Statistical Analysis

The SPSS and GraphPad Software were used for the statistical analysis. Data are represented as mean ± SD. All experiments were carried out in triplicates. Student’s *t*-test was used to evaluate the significance of differences. The Pearson rank correlation analysis was applied to examine the correlations between variables. The log-rank test and Kaplan–Meier method were used to analyze overall survival. *p* < 0.05 was considered statistically significant.

## 3. Results

### 3.1. SMYD2 Is Overexpressed in HCC and Correlates with Poor Prognosis

**Figure 1:**
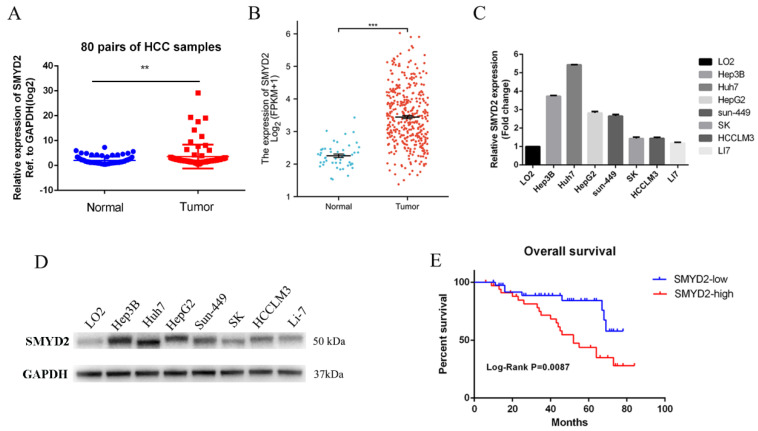
SMYD2 is overexpressed in HCC and is associated with unfavorable prognosis. (**A**) The expression level of SMYD2 in 80 HCC specimens and paired adjacent liver specimens was examined by RT-qPCR. ** *p* < 0.01. (**B**) The expression of SMYD2 in TCGA datasets. *** p < 0.001.(**C**) The mRNA level of SMYD2 in seven HCC cell lines and LO2. (**D**) The Protein level of SMYD2 in seven HCC cell lines and LO2. (**E**) Kaplan–Meier analysis of overall survival time of HCC patients based on SMYD2 expression (*n* = 74).

To investigate the function of SMYD2 in hepatocellular carcinoma, we detected the SMYD2 expression level in 80 pairs of HCC tissues and their paired normal specimens using RT-qPCR. SMYD2 mRNA level was overexpressed in HCC tissues (Figure 1A). Additionally, the TCGA database also validated that SMYD2 was overexpressed in HCC specimens (Figure 1B). Next, we detected the mRNA and protein expression of SMYD2 in seven HCC cell lines and normal hepatocytes (LO2). Consistent with the higher expression in HCC tissues, SMYD2 was overexpressed in the HCC cell lines (Figure 1C,D). To investigate whether the overall survival rate was correlated with the expression of SMYD2, we divided 74 HCC patients into two groups (low expression group and high expression group) based on the median value. We analyzed the relationship between the clinicopathologic factors of the HCC patients and their SMYD2 expression and found that SMYD2 is positively correlated with tumor number, tumor size, and age (Table 1). Moreover, the results indicated that patients with low SMYD2 expression had a better overall survival time (Figure 1E).

### 3.2. Knockdown of SMYD2 Inhibited the Proliferation of HCC Cells

**Figure 2:**
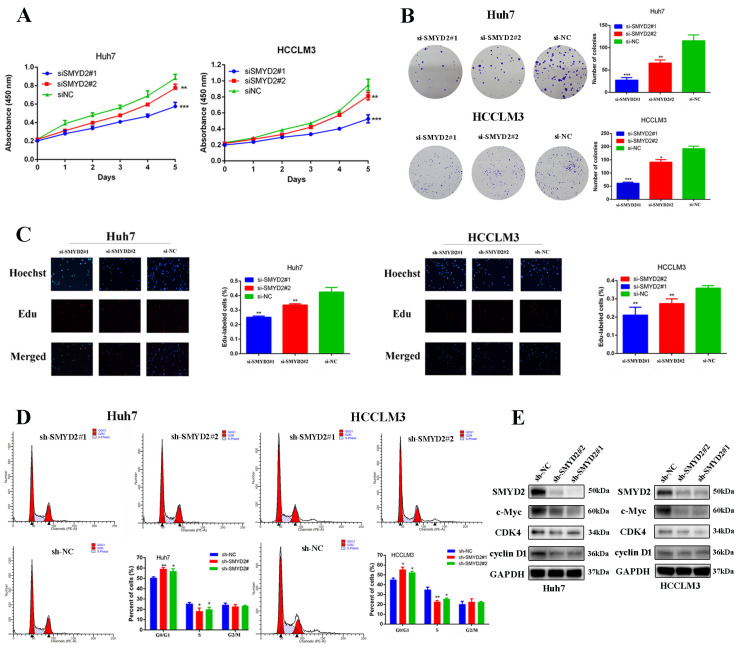
SMYD2 promotes HCC cell proliferation. (**A**) CCK-8 assay was used to examine cell growth after SMYD2 knockdown. (**B**) Colony formation assay was conducted in siRNA-transfected HCCLM3 cells and Huh7 cells (scale bar: 200 μm). Representative images (left) and relative colony numbers (right) are presented. (**C**) Representative images of EdU assays (red signal). (**D**) Cell cycle distribution was examined by flow cytometry. (**E**) Alternation of SMYD2 knockdown on G1/S cell-cycle-related proteins in the indicated cells was detected. Data are represented as mean ± SD. Student’s *t*-test was used to analyze the data. * *p* < 0.05, ** *p* < 0.01, *** *p* < 0.001.

To further determine the roles of SMYD2 in HCC progression, we knocked down the expression of SMYD2 in Huh7 and HCCLM3 cell lines with two different siRNAs (Supplemental Figure S1). After SMYD2 knockdown, we found the colony formation and proliferation of Huh7 and HCCLM3 cells were significantly inhibited (Figure 2A,B). Next, we conducted 5-ethynyl-2′-deoxyuridine (EdU) assays to further confirm the function of SMYD2 in HCC proliferation and found HCC cell proliferation was distinctly impaired after SMYD2 knockdown (Figure 2C). We then discovered that SMYD2 knockdown caused G0/G1 arrest of HCC cells, which is consistent with the results of its function in cell viability and colony formation assay (Figure 2D). To verify the findings, we generated stably transfected SMYD2 knockdown Huh7 and HCCLM3 cells using shRNA and then examined cell cycle proteins and found that SMYD2 knockdown inhibited the expression of c-Myc, CDK4, and cyclinD1 (Figure 2E).

### 3.3. SMYD2 Knockdown Suppressed Glutamine Metabolism by Silencing GLS1

**Figure 3:**
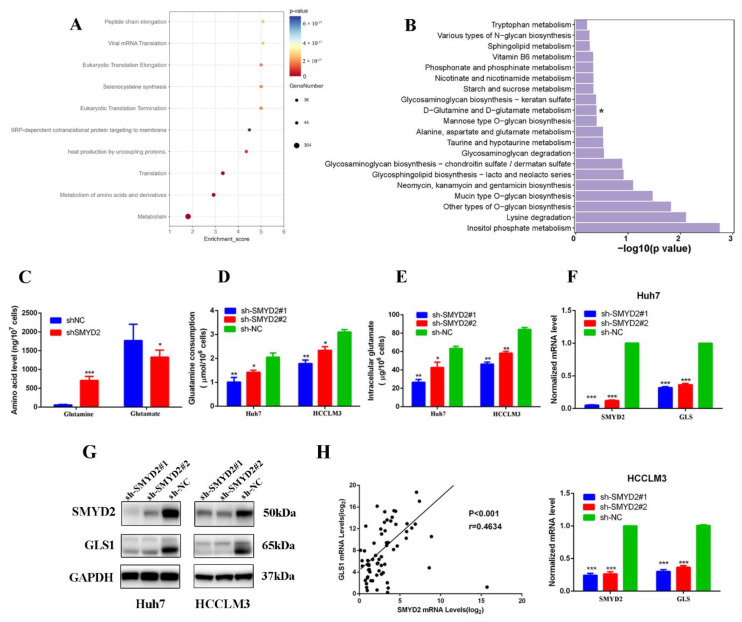
SMYD2 enhances glutamine metabolism in HCC cells. (**A**) Bubble diagram of the top ten enriched Reactomes. (**B**) KEGG pathway analysis of SMYD2-regulated genes. * *p* < 0.05. (**C**) Intracellular glutamine and glutamate of Huh7 shSMYD2 and sh-NC cells were analyzed by LC-MS/MS. Data are from three independent experiments. Student’s *t*-test was used to analyze the data.* *p* < 0.05, *** *p* < 0.001. (**D**) Glutamine consumption was detected in HCCLM3 and Huh7 cells expressing SMYD2-sh1 or sh-NC using the colorimetric method. Student’s *t*-test was used to analyze the data. * *p* < 0.05, ** *p* < 0.01.(**E**) Intracellular glutamate production was measured in HCCLM3 and Huh7 cells expressing SMYD2-sh1 or sh-NC using the colorimetric method. Data are from three represented as mean ± SD. * *p* < 0.05, ** *p* < 0.01. The data were analyzed using Student’s *t*-test. (**F**) The mRNA expression of GLS1 in Huh7 and HCCLM3 cells expressing shNC, sh-SMYD2#1, or shSMYD2#2. *** *p* < 0.001 (**G**) The protein levels of SMYD2 and GLS1 were examined in Huh7 and HCCLM3 cells expressing SMYD2-sh1 or sh-NC. (**H**) The correlation between SMYD2 and GLS1 in 74 HCC tissues was analyzed in a scatter plot analysis.

In order to investigate the global changes in SMYD2-dependent transcriptome, a genome-wide RNA sequencing analysis was carried out in SMYD2-silenced cells compared with scrambled cells. The reactome pathway analysis indicated that SMYD2 expression is correlated with the metabolism of amino acids (Figure 3A). Meanwhile, several pathways were analyzed through the Kyoto Encyclopedia of Genes and Genomes analysis. Intriguingly, we found the SMYD2 expression correlated to the pathways which were involved in glutamine metabolism, suggesting the pivotal role of SMYD2 in glutamine metabolism regulation (Figure 3B). As abnormal glutamine metabolism is crucial in HCC, we wondered whether SMYD2 could regulate glutamine metabolism in HCC. The intracellular amino acid levels were detected by liquid chromatography–tandem mass spectrometry (LC-MS/MS). We found the contents of glutamine were significantly increased by SMYD2 knockdown (Figure 3C). Moreover, the SMYD2-silenced cells consumed less glutamine (Figure 3D). Consistent with the inhibition of glutamine consumption, the level of intracellular glutamate was also lower in SMYD2-silenced cells (Figure 3E). Next, we investigated the biological role of SMYD2 in modulating glutamine metabolism. Interestingly, the glutaminase (GLS1) expression was downregulated in the indicated cells transfected with SMYD2-sh1 or SMYD2-sh2 (Figure 3F). Subsequent results validated that protein levels of GLS1 were also decreased in SMYD2 knockdown cells (Figure 3G). Moreover, scatter plot analysis showed that SMYD2 was positively correlated with GLS1 (*p* < 0.01, r = 0.4634) (Figure 3H). These results indicate that SMYD2 affects glutamine metabolism via the upregulation of GLS1.

### 3.4. SMYD2 Enhances c-Myc Stability at the Post-Transcription Level

**Figure 4:**
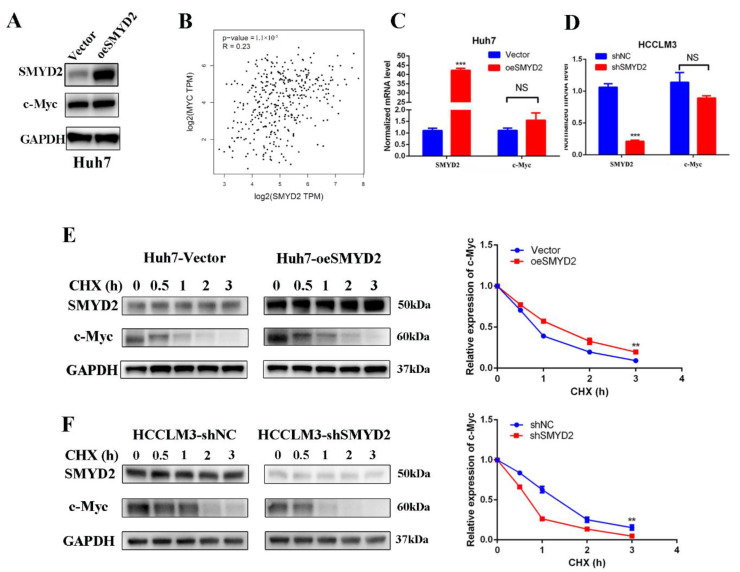
SMYD2 stabilizes c-Myc in HCC cells at the post-transcription level. (**A**) The protein level of c-Myc in the SMYD2-overexpressing Huh7 cells. (**B**) Scatter plot shows the lack of correlation between SMYD2 and c-Myc. (**C**,**D**) The mRNA level of c-Myc in SMYD2 overexpressed (**C**) and SMYD2 knockdown (**D**) HCC cell lines. Data are represented as mean ± SD. The data were analyzed by Student’s *t*-test. GAPDH served as the internal control. NS: not significant. *** *p* < 0.001. (**E**) Effect of cycloheximide (10 μg/mL) on c-Myc in SMYD2 overexpressed HCC cells at several time points. The expression of SMYD2 and c-Myc was examined by Western blot (**left**) and semi-quantification (**right**).** *p* < 0.01. (**F**) Effect of cycloheximide (10 μg/mL) in SMYD2 knockdown HCCLM3 cells in the indicated times. ** *p* < 0.01.

Next, we investigated the underlying mechanism of how SMYD2 upregulates the expression of GLS1. Recent studies have suggested that MYC may regulate GLS1 to affect the glutaminolysis pathways [17,24]. Myc is an oncogene that is dysregulated in many tumors and affects cancer cells’ proliferation, their response to stress, and metabolism reprogramming [25]. Therefore, we presume that SMYD2 might promote HCC cell proliferation and reprogram glutamine metabolism via c-Myc. Surprisingly, SMYD2 knockdown significantly inhibited the protein expression of c-Myc (Figure 2E). Moreover, the ectopic expression of SMYD2 in Huh7 cells resulted in an increasing trend in Huh7 cells (Figure 4A). Then, we investigated whether SMYD2 affects the c-Myc expression at the post-transcriptional or the transcriptional level. Data from The Cancer Genome Atlas (TCGA) database suggested a lack of correlation between the c-Myc and SMYD2 mRNA expression levels (Figure 4B). Then, we performed RT-qPCR to measure the mRNA level of c-Myc in SMYD2 knockdown and SMYD2 overexpressed cells and found there are no significant differences in c-Myc expression between these cells (Figure 4C,D). Based on these results, we suggest that SMYD2 may regulate c-Myc at the post-translational level. Thus, we used cycloheximide (CHX) to treat HCC cells at indicated time points. As shown in Figure 4E, pretreatment with CHX led to a prolonged c-Myc half-life in the SMYD2 overexpressed Huh7 cells. In contrast, knockdown SMYD2 in HCCLM3 cells caused a greater degradation of c-Myc (Figure 4F).

### 3.5. SMYD2 Stabilized c-Myc by Regulating the Ubiquitin-Proteasome System

**Figure 5:**
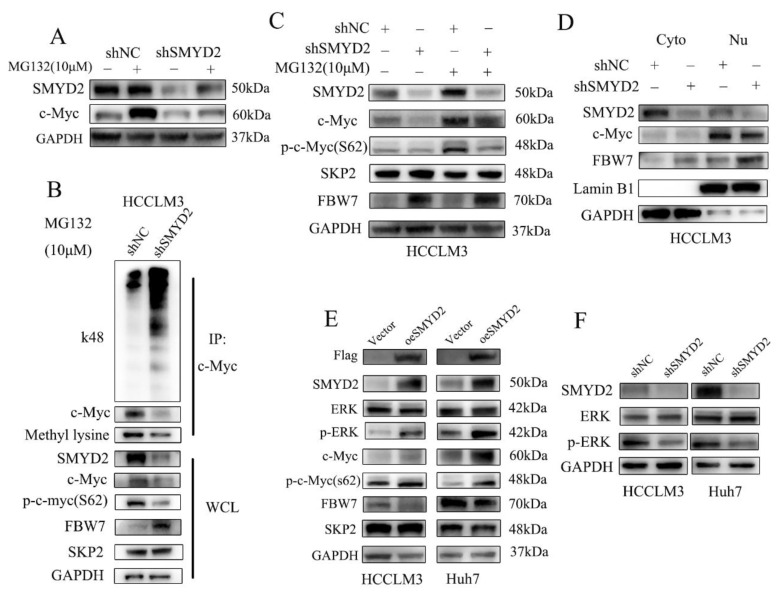
SMYD2 protects c-Myc protein from ubiquitination and degradation. (**A**) The effects of MG132 on c-Myc expression in SMYD2 knockdown HCCLM3 cells were determined using Western blot. The indicated cells were treated with MG132 (10 μM) for 4–6 h. (**B**) Immunoprecipitation (IP) was performed to detect the methylation and K48 polyubiquitination level of c-Myc in HCCLM3-shSMYD2 and HCCLM3-shNC cells. After exposing to MG132 (10 μM) for 4–6 h, extracts were used to IP with c-Myc antibody. (**C**) Effects of MG132 on SKP2, p-c-Myc(S62), FBW7 and c-Myc in SMYD2 knockdown cells. (**D**) Cytoplasmic and nuclear FBW7 and c-Myc expression were examined in HCCLM3-shSMYD2 and HCCLM3-shNC cells. Lamin B1 and GAPDH were selected as internal standards. Nu: nucleus. Cyto: cytoplasm. (**E**) Influence of overexpressed SMYD2 on the indicated proteins in HCCLM3 and Huh7 cells. (**F**) The effect of SMYD2 knockdown on p-ERK in indicated cells.

The oncogenic transcription factor c-Myc is reported as a short-lived protein that is usually degraded by the ubiquitin–proteasome pathway [26]. Therefore, we examined whether SMYD2 regulated c-Myc in HCC cells via proteasome-dependent-degradation. We found that treating HCC cells with proteasome inhibitor MG132 could reverse SMYD2 knockdown-induced c-Myc downregulation (Figure 5A). These findings indicated that SMYD2 may regulate c-Myc via proteasomal degradation. It is well established that SMYD2 could methylate nonhistone proteins [22]. Next, we explored whether SMYD2 methylates c-Myc. Figure 5B shows the knockdown of SMYD2 could downregulate c-Myc methylation in HCCLM3 cells. To our surprise, SMYD2 knockdown promoted the k48 ubiquitination level of c-Myc. These results indicated that SMYD2-mediated methylation is crucial for inhibiting c-Myc ubiquitination and degradation.

MYC S62 can be phosphorylated by JNK and ERK, which stabilized MYC protein [27]. In our study, we found the phosphorylation of Ser62 of MYC could be reversed by treating cells with MG132 (Figure 5C). It has been reported that E3 ubiquitin ligases, such as SKP2 and FBW7, could degrade c-Myc. Surprisingly, we found that SMYD2 knockdown could upregulate the expression of FBW7 (Figure 5C). Then, we investigated nuclear and cytoplasmic proteins of HCC cells by Western blot. Interestingly, FBW7 was upregulated both in the cytoplasm and nuclei of the SMYD2 knockdown HCCLM3 cells (Figure 5D). Conversely, the overexpression of SMYD2 enhanced the phosphorylation of Ser62 in MYC and downregulated FBW7 expression but not SKP2 in the indicated HCC cells (Figure 5E).

Studies have shown that E3 ubiquitin ligases FBW7 could be phosphorylated by ERK kinase [28]. Additionally, the MAPK/ERK pathway can regulate the phosphorylation of c-Myc (S62) [29]. So we presume that SMYD2 might regulate the FBW7 and c-Myc expression via the ERK pathway. As expected, SMYD2 overexpression upregulated the phosphorylation of ERK in HCCLM3 and Huh7 cells (Figure 5E). In contrast, SMYD2 knockdown downregulated phosphorylation of ERK in the indicated HCC cells (Figure 5F). Next, we further explored whether c-Myc involves in SMYD2-regulated HCC cells’ proliferation. We found FBW7 depletion restored the proliferation of HCCLM3 cells and the protein level of c-Myc which was induced by SMYD2 knockdown (Supplementary Figure S2A,B). Moreover, we pretreated SMYD2 overexpressed HCC cells with 10058-F4, a specific c-Myc inhibitor, and c-Myc siRNA to effectively suppressed their c-Myc expression. CCK-8 assays demonstrated that suppressing c-Myc by inhibitor or siRNA can partially inhibit the enhanced proliferation induced by SMYD2 overexpression (Supplementary Figure S2C,D).

In summary, these data indicated that SMYD2 stabilized c-Myc by enhancing its methylation which inhibited its ubiquitin-dependent degradation. Additionally, our results showed that c-Myc plays an important role in SMYD2-mediated HCC proliferation.

### 3.6. SMYD2 Upregulated GLS1 Expression through c-Myc

**Figure 6:**
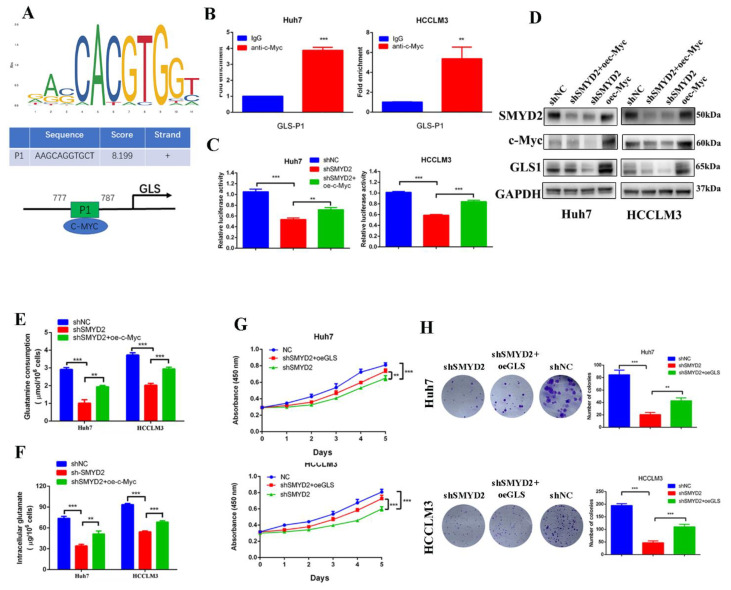
SMYD2 upregulates the expression of GLS1 through c-Myc. (**A**) Schematic illustration shows the potential c-Myc-binding site. (**B**) The enrichment of c-Myc on the GLS1 promoter was examined using ChIP-qPCR assay. The presented data were analyzed by Student’s *t*-test. ** *p* < 0.01, *** *p* < 0.001. (**C**) Luciferase reported assay to determine the GLS1 promoter activity in indicated HCC cells. Student’s *t*-test was used to analyze the data. Data are represented as mean ± SD. ** *p* < 0.01, *** *p* < 0.001.(**D**) Western blotting was conducted to examine the protein level of c-Myc, GLS1, and SMYD2. (**E**,**F**) The glutamine consumption and intracellular glutamate level were detected in indicated HCC cells. ** *p* < 0.01, *** *p* < 0.001. Data are represented as mean ± SD. Data are from three independent experiments. Student’s *t*-test was used to analyze the data. (**G**) Cell viability was examined by CCK-8 assay. ** *p* < 0.01, *** *p* < 0.001. Student’s *t*-test was conducted to analyze the data. (**H**) Colony formation of the indicated HCC cells. ** *p* < 0.01, *** *p* < 0.001. Data are represented as mean ± SD. Student’s *t*-test was used to analyze the data.

Based on the above findings, we presumed that SMYD2 might regulate the expression of GLS1 by c-Myc. The JASPAR database showed GLS1 promoter region contains potential binding sites for c-Myc (Figure 6A). Then, we conducted the chromatin immunoprecipitation (ChIP) assay to demonstrate c-Myc could bind to the GLS1 promoter (Figure 6B). Furthermore, overexpressing c-Myc could rescue the SMYD2-induced GLS1 promoter suppression (Figure 6C). Meanwhile, the protein levels of GLS1 and c-Myc were downregulated when SMYD2 knockdown, but the GLS1 expression could be partly restored by overexpressing c-Myc (Figure 6D). Additionally, overexpression of c-Myc rescued shSMYD2-induced glutamine consumption inhibition and intracellular glutamate levels downregulation in HCC cells (Figure 6E,F).

Due to the critical role of glutamine metabolism in tumor progression, we investigated whether GLS1 was the downstream effector of SMYD2. Colony formation and CCK-8 assays indicated that GLS1 overexpression partly rescued the SMYD2 knockdown-induced inhibition of HCC cell proliferation (Figure 6G,H). In summary, these results indicated that SMYD2 upregulates GLS1 through c-Myc and GLS1 is an important downstream effector in SMYD2-regulated HCC proliferation.

### 3.7. SMYD2 Promotes HCC Tumor Growth and Enhances Their Chemoresistance to Sorafenib

**Figure 7:**
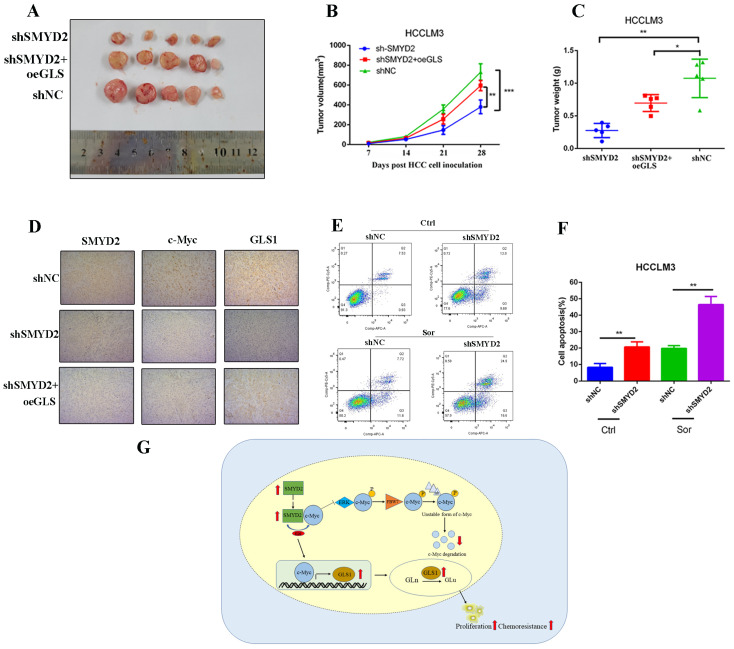
SMYD2 promotes HCC tumor growth and enhances sorafenib chemoresistance in HCC. (**A**) Photographic image of the subcutaneous tumors. (**B**,**C**) Tumor volumes (**B**) and weight (**C**) were measured. The data are from five mice per group and presented as mean ± SD. The data were analyzed with Student’s *t*-test. * *p* < 0.05, ** *p* < 0.01, *** *p* < 0.001.(**D**) IHC analysis was conducted to examine the expression of SMYD2, c-Myc, and GLS1(scale bar: 100 μm; magnification: ×200). (**E**,**F**) Cells were incubated with 5 μM sorafenib (Sor) for 24 h. Representative images of flow cytometry analysis (**E**) and quantification (**F**) are presented. Data are represented as mean ± SD. ** *p* < 0.01. The data were analyzed by Student’s *t*-test. (**G**) Schematic representation shows that SMYD2 upregulates GLS1 expression through the SMYD2/c-Myc cascade, thereby regulating glutamine metabolism and promoting HCC growth. Abbreviations: Gln, glutamine; Glu, glutamate.

Then, we conducted in vivo experiments to investigate the function of SMYD2 in HCC. We subcutaneously injected SMYD2-knockdown HCCLM3 cells with or without GLS1 overexpression into BALB/c nude mice. After four weeks, we discovered that GLS1 overexpression could promote tumor growth in the SMYD2-knockdown model (Figure 7A–C). Moreover, immunohistochemistry indicated that the expressions of c-Myc and GLS1 were downregulated in SMYD2-deficient HCCLM3 xenografts, and c-Myc expression could be largely rescued by GLS1 overexpression (Figure 7D).

Currently, sorafenib is the first-line drug for HCC patients’ treatment. Studies have shown that glutamine metabolism reprogramming plays a crucial role in generating sorafenib resistance in HCC cells [30,31]. Therefore, we investigated whether inhibiting glutamine metabolism could sensitize SMYD2-deficient HCCLM3 cells’ response to sorafenib. Our results showed SMYD2-deficient HCC cells underwent more apoptosis when treated with sorafenib (Figure 7E,F). In summary, these data suggest that SMYD2 promotes HCC cells’ growth and enhances their chemoresistance to sorafenib.

## 4. Discussion

SMYD2 has been demonstrated to play pivotal roles in multiple tumors, such as acute lymphoblastic leukemia [32], breast cancer [33], and gastric cancer [34]. It has been reported that SMYD2 predicts poor prognosis in HCC [35]. However, the role of SMYD2 in HCC remains undefined. In the current study, we showed SMYD2 is overexpressed in hepatocellular carcinoma and correlates with unfavorable clinical outcomes. The downregulation of SMYD2 dramatically inhibits the proliferation of HCC cells. Furthermore, we verified SMYD2 is a stabilizer of c-Myc that further promotes c-Myc expression in HCC. Mechanistically, SMYD2 enhances glutamine metabolism via the c-Myc/GLS1 axis. We show that GLS1 is the downstream effector of SMYD2 and is required for SMYD2-regulated HCC tumor growth. Silencing SMYD2 also sensitized HCC cells to sorafenib. Therefore, these results indicate that SMYD2 can be a potential therapeutic target in HCC.

Glutamine is involved in the macromolecular synthesis, signaling, and energy formation [36]. Recent findings have revealed that glutamine metabolism is reprogrammed in multiple solid tumor progressions, including HCC [30,37]. In our research, we found that SMYD2 regulates glutamine metabolism through GLS1. Glutaminase (GLS1) catalyzes the glutamine to glutamate, which further enters into the TCA cycle. It has been revealed that GLS exists as two isozymes named GLS1 and GLS2. An accumulation of evidence has shown that GLS1 is overexpressed in multiple malignant and may serve as an oncogene, while GLS2 acts as a tumor suppressor [15,38]. GLS1 can participate in tumor progression and migration and is correlated with poor clinical outcomes [17,24,39]. Silencing GLS1 dramatically inhibits the invasion and proliferation of many tumors [40]. Our results suggest that GLS1 may be a potential therapeutic target in hepatocellular carcinoma. Given that glutamine is an important carbon source for anabolic processes and energy production, we conjectured that the SMYD2-regulated glutamine metabolism reprogramming by GLS1 is critical for HCC growth. Our research verifies that GLS1 overexpression can restore the suppression of HCC growth induced by SMYD2 knockdown. These data demonstrated that GLS1 acts as a downstream effector of SMYD2-mediated HCC growth.

The correlation between c-Myc and many important cellular processes such as DNA replication, and macromolecule biosynthesis, has been well studied and our results provide more explanations of how c-Myc reprograms glutamine metabolism [37,41]. It is reported c-Myc could regulate GLS in response to nutrient stress [24]. Apart from methylating histone H3K4 and H3K36, SMYD2 also regulates the methylation of diverse nonhistone substrates [19,42]. Here, we showed that c-Myc methylated by SMYD2 further stabilizes c-Myc protein by inhibiting its interaction with FBW7 and by disturbing ubiquitin–proteasome-dependent degradation.

Then, we investigated whether SMYD2 regulates the expression of GLS1 through c-Myc. The luciferase reporter and ChIP-qPCR assays demonstrated that c-Myc transcriptional activating GLS1 promoter. We also demonstrated the role of c-Myc in reprogramming glutamine metabolism in HCC cells. Moreover, sorafenib is the first-line drug for the treatment of HCC. Recent studies showed that cancer cells prefer using glutamine for lipid biosynthesis. It raises the question of whether deregulating glutamine metabolism enhances sorafenib chemoresistance. Interestingly, our results validated that SMYD2-deficient HCC cells are more sensitive to treatments with sorafenib.

Taken together, in the current study, we demonstrated that SMYD2 participates in the progression of hepatocellular carcinoma and correlates with poor clinical outcomes. SMYD2 reprograms glutamine metabolism via the c-Myc/GLS1 axis (Figure 7G). Therefore, SMYD2 may be a potent therapeutic target, as well as a promising prognostic biomarker in HCC.

## Figures and Tables

**Table 1:** Correlation between SMYD2 expression and clinicopathological features in HCC patients.

Variables	SMYD2 Expression	*p*-Value
	Low High	
Age		
≤50 years	20 11	0.034
>50 years	17 26	
Preoperative AFP level		
≤400 ng/ml	21 22	0.814
>400 ng/ml	16 15	
Sex		
Male	23 21	0.636
Female	14 16	
Histopathological grading		
Well + moderately	17 19	0.642
Poorly	20 18	
Tumor size		
≤5 cm	25 13	0.005
>5 cm	12 24	
Tumor number		
Single	28 16	0.004
Multiple	9 21	
Tumor stage		
I–II	18 17	0.816
III–IV	19 20	
HBV		
Negative	27 24	0.451
Positive	10 13	

## Data Availability

The datasets used and analyzed during the current study are available from the corresponding author upon reasonable request.

## Supplementary Materials

The following supporting information can be downloaded at: https://www.mdpi.com/article/10.3390/cells12010025/s1, Figure S1: Validation of SMYD2 knockdown in HCC cell lines; Figure S2: c-Myc participates the SMYD2-mediated HCC progression. Table S1: The sequence of primers used in this study. Table S2: The sequence of ChIP-qPCR primers. Table S3: The sequences of siRNAs. Table S4: Primary antibodies used in the study.
