# Supplementary material for: SMYD2 Promotes Hepatocellular Carcinoma Progression by Reprogramming Glutamine Metabolism via c-Myc/GLS1 Axis
Source: Cells. 2022 Dec 21;12(1):25. doi: 10.3390/cells12010025 (PMC9818721; doi:10.3390/cells12010025)
Supplement: Supplementary file 1 [file cells-12-00025-s001.zip › cells-2061692-supplementary.pdf]

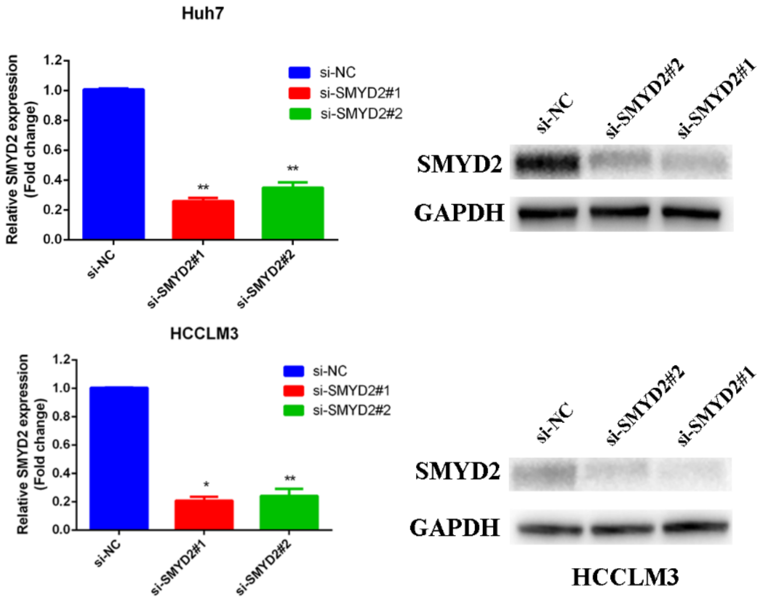

Supplementary Figure S1 Validation of SMYD2 knockdown in HCC cell lines. The efficiency of SMYD2 knockdown in Huh7 and HCCLM3 cells was detected by qRT-PCR and Western blot.

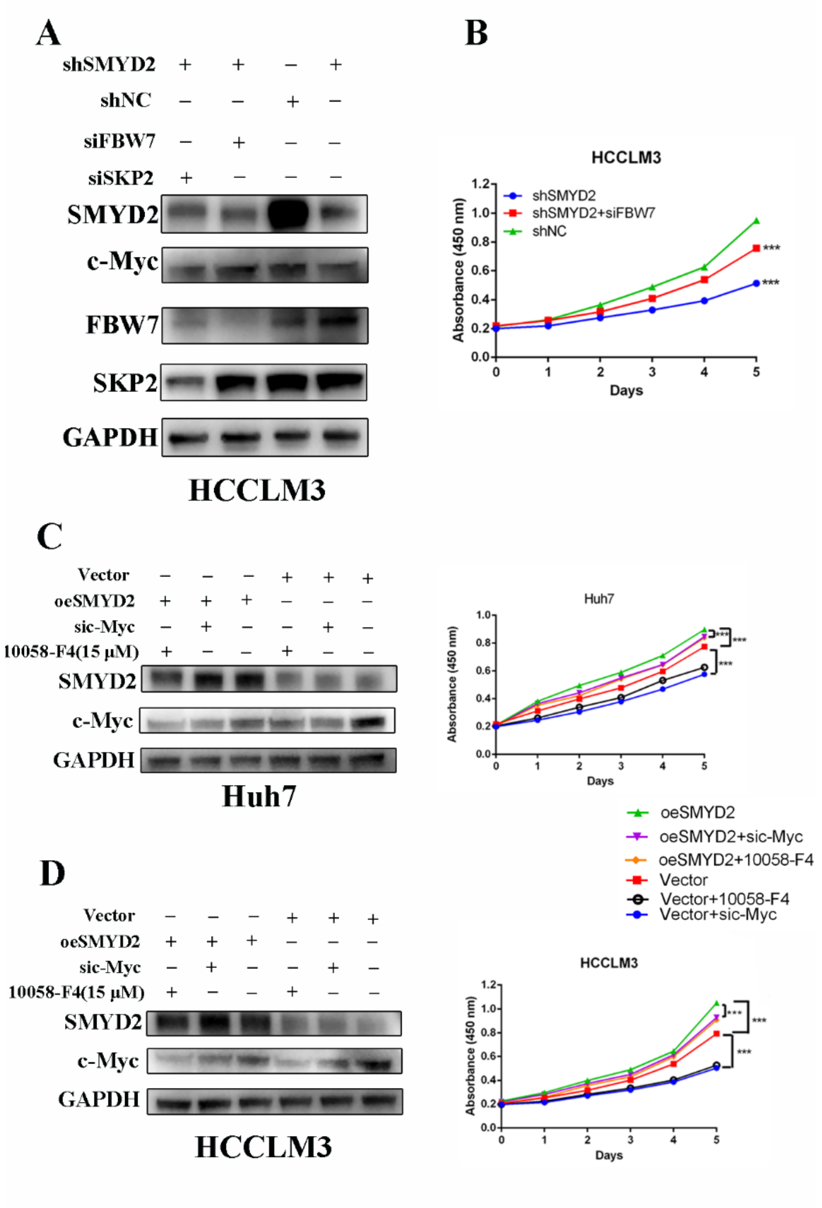

Supplementary Figure S2 c-Myc participates the SMYD2-mediated HCC progression. **A** The expression levels of c-Myc, SMYD2, SKP2 and FBW7 after treat HCCLM3-shSMYD2 cells with FBW7 or SKP2 siRNAs. **B** The proliferation of indicated HCCLM3 cells was evaluated by CCK-8 assays. **C** The inhibition efficiency after treat Huh7-pcDNA and HCCLM3-pcDNA-SMYD2 cells with a specific c-Myc inhibitor 10058-F4 (15μM) or c-Myc siRNA. **D** CCK-8 assay for indicated Huh7 and HCCLM3 cells were evaluated. Data are represented as mean ± SD. ***p<0.001. Student's *t* test was used to analyze the data.

Table S1 The primers used in qRT-PCR are listed.

	**qRT-PCR Primers**	
**Gene**	**Forward Sequence (5′-3′)**	**Reverse sequence (5′-3′)**
SMYD2	CCTGACAATGATAGCCTCGTAG	GTCCTTGGTGGTACACTCCT
GAPDH	GGAGCGAGATCCCTCCAAAAT	GGCTGTTGTCATACTTCTCATGG
c-MYC	GGCTCCTGGCAAAAGGTCA	CTGCGTAGTTGTGCTGATGT
GLS1	TCCCCAAGGACAGGTGGAAT	GAGGTGTGTACTGGACTTGGT

Table S2 The primers for the indicated promoters are as follows.

	**ChIP-qPCR Primers**	
**Gene**	**Forward Sequence(5′-3′)**	**Reverse sequence(5′-3′)**
GLS1-P1	GCCTGTAATCCCAGCAATTT	GGGGGTTGGTGTGTTGTTAC

Table S3 Sequences of siRNAs were as follows.

**Gene**	**Forward**	**Reverse**
SMYD2#1	GAAAUGACCGGUUAAGAGATT	UCUCUUAACCGGUCAUUUCTT
SMYD#2	GAAUGACCGGUUAAGAGATT	UCUCUUAACCGGUCAUUCTT
siNC	UUCUCCGAACGUGUCACGUTT	ACGUGACACGUUCGGAGAATT
c-Myc	CAGAAAUGUCCUGAGCAAUTT	AUUGCUCAGGACAUUUCUGTT
FBW7	CCAUGCAAAGUCUCAGAAUTT	AUUCUGAGACUUUGCAUGGTT
SKP2	GCCUAAGCUAAAYCGAGAGAATT	UUCUCUCGAUUUAGCUUAGGCTT

Table S4 Primary antibodies used in this study.

Antibody	Company (Cat. No.)	Working	Concentration
SMYD2	Proteintech (21290-1-AP)	WB: 1/1000	IHC:1/20
GLS1	abcam (ab156876)	WB: 1/1000	IHC:1/100
CDK4	Abcam (ab199728)	WB: 1/2000	
Cyclin D1	Abcam (ab134175)	WB: 1/2000	
c-Myc (phospho-S62)	Abcam (ab185656)	WB: 1/1000	
Methylated Lysine	Abcam (ab23366)	WB: 1/1000	
Lamin B1	Abcam (ab229025)	WB: 1/1000	
FBW7	Proteintech (28424-1-AP)	WB: 1/3000	
Flag	Abcam (ab205606)	WB: 1/3000	
SKP2	Abcam (ab183039)	WB: 1/200	
ERK	Abcam (ab184699)	WB: 1/5000	
p-ERK	Abcam (ab32538)	WB: 1/1000	
Ubiquitin (linkage-specific K48)	Abcam(ab140601)	WB: 1/1000	
GAPDH	Proteintech (10494-1-AP)	WB: 1/5000	
c-Myc	Cell Signaling (#18583)	WB: 1/1000	ChIP: 1/100
c-Myc	Proteintech (67447-1-IG)	IHC: 1/100	
